# Response to ‘Non-complicit: Revisiting Hans Asperger’s Career in Nazi-era Vienna’

**DOI:** 10.1007/s10803-019-04106-w

**Published:** 2019-06-13

**Authors:** Herwig Czech

**Affiliations:** 1 Department of Ethics, Collections and History of Medicine, Medical University of Vienna, Währinger Straße 25, 1090 Vienna, Austria; 2 Present Address: Institute for the History of Medicine and Ethics in Medicine, Charité – University Medicine Berlin, Thielallee 71, 14195 Berlin, Germany

**Keywords:** Asperger syndrome, Hans Asperger, National socialism, Nazi ‘euthanasia, Nazi-era Vienna, Therapeutic pedagogy (Heilpädagogik)

## Abstract

In her recent paper ‘Non-complicit: Revisiting Hans Asperger’s Career in Nazi-era Vienna,’ Dean Falk claims to refute what she calls ‘allegations’ about Hans Asperger’s role during National Socialism documented in my 2018 paper ‘Hans Asperger, National Socialism, and “race hygiene” in Nazi-era Vienna’ and Edith Sheffer’s book ‘Asperger’s Children.’ Falk’s paper, which relies heavily on online translation software, does not contain a single relevant piece of new evidence, but abounds with mistranslations, misrepresentations of the content of sources, and basic factual errors, and omits everything that does not support the author’s agenda of defending Hans Asperger’s record. The paper should never have passed peer review and, in view of the academic credibility of all parties concerned, it should be retracted.

In her paper ‘Non-complicit: Revisiting Hans Asperger’s Career in Nazi-era Vienna’, evolutionary anthropologist Dean Falk claims to refute what she calls ‘allegations’ raised against Asperger (Czech 2018; Sheffer 2018) ‘with newly translated and chronologically-ordered information that takes into account Hitler’s deceptive “halt” to the T4 euthanasia program in 1941.’ She states that ‘[i]t is highly unlikely that Asperger was aware of the T4 program when he referred Herta Schreiber to Vienna’s child “euthanasia” facility Am Spiegelgrund or when he mentioned that institution 4 months later on the medical chart of another (unrelated) girl, Elisabeth Schreiber’ (Falk 2019). However, the ‘newly translated’ information presented to ‘exonerate’ Asperger appears largely irrelevant to the questions at hand, while the various arguments put forward are characterized by fundamental factual errors, misleading quotes, mistranslations of German language sources (the author had to rely on online translation tools), and a refusal to seriously engage with the evidence presented in my paper by omitting everything that does not support the author’s manifest agenda of defending Hans Asperger’s record.

There are, however, two points on which I agree with Falk. First, as stated in my paper, I see no reason to consider the validity of Asperger’s scholarship as tainted per se by its historical context and Asperger’s concessions towards the Nazi regime (Czech 2018, p. 32). Edith Sheffer’s attempt to define the concept of autistic psychopathy as intrinsically tarnished by an affinity to National Socialist ideology is conceptually and methodologically weak, as she exaggerates the importance attributed at the time to the concept of *Gemüt* (in an approximate translation: ‘disposition’ or ‘soul’), which supposedly defines what she calls ‘Nazi psychiatry.’ In reality, psychiatry during National Socialism is much better characterized by the concept of ‘life unworthy of living,’ while *Gemüt* was only one of several personality traits discussed at the time, moreover one that was not particularly important to Asperger and his definition of ‘autistic psychopathy’ (Asperger 1944a). Second, I have strengthened the case for Asperger’s chronological priority over Leo Kanner, showing that the 1938 paper on abnormal children—now provided by Falk in full translation, albeit with errors^1^—was available early on to Leo Kanner in Baltimore.

Due to numerous errors and/or misunderstandings regarding the historical facts and the original sources, Falk’s paper does a disservice to the legitimate and necessary debate surrounding Asperger’s biography during the Nazi period. Throughout her paper, she wrongly attributes the Viennese Spiegelgrund facility—where Herta Schreiber and Elisabeth Schreiber along with hundreds of other children were killed in the so called ‘child euthanasia’ program—to ‘Aktion T4,’ the killing of psychiatric patients in six centralized killing centers equipped with gas chambers and crematoria. This severely compromises the entire argument around Hitler’s “halt” to Aktion T4 and the presented chronology of transferals supposed to prove Asperger’s ignorance of the dangers faced by his patients at Spiegelgrund. It also raises the question of how such a fundamental error could have passed peer review. The reference to Hitler’s ‘euthanasia halt’ of August 24, 1941 is based on a triple misunderstanding—first, that Spiegelgrund was part of T4 (which it was not), second, that Hitler’s order was made public (which it was not; misleadingly, Falk calls it a ‘Nazi public relations ploy’), and third, that this order (or Bishop Galen’s sermon that prompted it) was a relevant potential source of information on Spiegelgrund for Asperger (which it was not). Falk also argues that after only four previous transferals from the University Children’s Clinic to Spiegelgrund with a deadly outcome, Asperger’s colleagues (and himself) did not yet have any reason to suspect foul play. This argument is based on false premises, notably that Asperger’s colleagues (including the director Franz Hamburger) were also in the dark regarding Spiegelgrund’s true purpose and that information on the murders started to spread only after the ‘halt’ order.

In reality, the fact that psychiatric patients were dying in great numbers under suspicious circumstances had already become widely known among the Viennese population in September 1940, months before Herta’s transferal (and Bishop Galen’s sermon), even leading to public protests (Klee 1985, p. 208–209). In November 1940, the *Völkischer Beobachter*, the official newspaper of the Nazi Party had to deny rumors that patients were being killed by poisoning and in gas chambers, referencing various Viennese institutions (Schödl 1940). Falk is correct in stating that transferals of children with disabilities from the Children’s Clinic to Spiegelgrund strongly increased after Hitler’s ‘halt’ order (by June 27, the day Asperger signed Herta’s transferal, 30 patients had died at Spiegelgrund; by October 27, the date of Elisabeth’s transferal note, the number had risen to 71).^2^ However, while this represents one potential way Asperger could have found out about Spiegelgrund’s purpose, it was certainly not the only source of information on the ‘euthanasia’ killings taking place practically before his eyes. Even if we assume that Asperger did not know the details of the ‘child euthanasia’ program at Spiegelgrund, he could not conceivably have been in the dark about the dangers faced by patients with mental disabilities at Steinhof psychiatric hospital, on the very premises of which Spiegelgrund was founded in July 1940. Spiegelgrund was not part of ‘Aktion T4’ (and therefore not affected by Hitler’s ‘halt’ order), but Asperger and his contemporaries could not be expected to have known this at the time, given the secrecy surrounding the killing operations and the fact that in Vienna one person—Erwin Jekelius—was in charge both of coordinating T4 and running Spiegelgrund. As it were, in June 1941 Asperger exposed Herta not only to the child euthanasia program at Spiegelgrund, but also to T4, which would only be suspended 2 months later.

The example of Anna Wödl, a nurse at the same General Hospital to which Asperger’s clinic belonged, illustrates how implausible it is that Asperger could have been in the dark about the dangers faced by his patients. Wödl, who had a son with a mental disability placed in an institution near Vienna, clearly recognized the threat he was under. By July 1940—almost a year before Asperger signed the transferal of Herta—she had gathered enough information to identify the Berlin official responsible for coordinating the T4 program (Herbert Linden) and to personally go see him and plead (ultimately unsuccessfully) for her son’s life (Fürstler and Malina 2003).

Are we really to assume that during this period not one parent of a child with a disability seen by Asperger shared similar worries with him? According to a report in Herta Schreiber’s Spiegelgrund file, her mother brought up her possible death as a relief.^3^ Are we really to assume that, while the mother at least considered the possibility that her child would die at Spiegelgrund, Asperger in good faith had no such suspicions? And how plausible is it that he never made the connection between the massive disappearance of patients (including children) during T4, the role of his former co-worker Erwin Jekelius (the city’s highest placed physician in charge of psychiatric patients, to whom inquiries regarding disappeared patients were regularly referred by his colleagues), and the Spiegelgrund, which was newly founded and placed under Jekelius’ leadership? That he never cared to ask what was going on, never suspected anything? Although we cannot know precisely what Asperger knew (or to what extent he was willing or able to take notice of what he certainly would have been able to find out had he wanted to), it stretches credulity that with all the information available to him in June 1941, he could have assumed in good faith that delivering Herta Schreiber into the hands of Erwin Jekelius, a man whom he had known for many years and whose position vis-à-vis ‘unworthy life’ was no secret, would mean that she would receive proper care rather than being killed one way or another.

Another misleading claim is that Elisabeth Schreiber, the second case mentioned in my paper, was not referred to Spiegelgrund on Asperger’s recommendation. Falk proposes an alternative—and erroneous—translation of Asperger’s note, claiming that the sentence ‘Am ehesten^4^ käme der “Spiegelgrund” in Frage’ should be translated as ‘most likely the “Spiegelgrund” came into question.’ This is a mistranslation on several counts: ‘am ehesten’ here does not express a probability, but the first preference among a set of given options; *käme* is not past tense (this would be *kam*), but a conditional form; and *infrage kommen* here clearly means ‘to be an option,’ not ‘to come into question.’ The most precise translation, therefore, is: ‘The “Spiegelgrund” would be the most appropriate option,’ or—in my shorter version—‘Spiegelgrund would be the best possibility.’ It is true—as I explain in my paper—that Elisabeth was first transferred to another institution before being sent to Spiegelgrund. Falk’s conclusion that the transferal was therefore not Asperger’s responsibility, however, is clearly disproven by the document reproduced in its entirety in my paper (and quoted by Falk), which states that Elisabeth’s transferal to Spiegelgrund less than 4 months later was arranged explicitly on the basis of Asperger’s assessment (Czech 2018, p. 22).

Falk’s argument (mentioned no less than five times in the paper and in Appendix 2) that Asperger was, because of his critical stance towards the Nazi regime, investigated over several years by ‘numerous Nazi officials’ (Falk 2019, p. 5), ‘the Nazis’ (p. 5), ‘the Nazi party’ (p. 10), and even ‘the Gestapo’ (Appendix 2, p. 7) is similarly misleading. In another passage, she even goes so far as to falsely attribute the claim of an ‘investigation by the Gestapo’ to me by combining it (within brackets) with a direct quote from my paper (Falk 2019, p. 5). In reality, as I explain in the referenced passage, the ‘preliminary investigation’ opened in 1938 was part of a general vetting operation of all public employees (which included university staff) undertaken after the ‘Anschluss’ in order to identify (and remove) Jewish and politically undesirable individuals (Czech 2018, p. 8–9). Such preliminary investigations were mandated in cases where the political reliability of a public employee was in question, which was the case here due to Asperger’s political past. As I also mention, this vetting was the responsibility of an official in Vienna’s local government, not the Gestapo. There is no evidence of any political trouble for Asperger after the procedure ended in his favor in June 1939. The only proven involvement of the Gestapo is that, when asked by the city’s personnel office for relevant information concerning Asperger, they responded that he had a clean record (Czech 2018, p. 8). The later ‘investigations’ were inquiries that routinely accompanied every promotion or career move, not just in the case of Asperger, but of everyone in a similar position. Again, the impression conveyed in Falk’s paper in this regard is a misrepresentation of the available sources, and of the historical context. The fact remains that the only source for Asperger’s alleged persecution by the Gestapo is Asperger himself. The 1957 speech presented by Falk as ‘new evidence’ does not contain any reference to the Gestapo (Asperger 1957), which only became part of Asperger’s narrative another 5 years later, in 1962 (Asperger 1962).

Another cornerstone of Falk’s argument is what she repeatedly refers to as ‘Asperger’s sustained campaign on behalf of disabled children’ (p. 2) and his ‘advocacy for disabled children’ (p. 6). Including variations, the paper and Appendix 2 contain nearly a dozen such references. This ‘campaign’ is another construction based on a misrepresentation of the sources. It ignores the fact that Asperger in the quoted passages did not refer to ‘disabled children,’ but to those with ‘mental abnormalities,’ who constituted the vast majority of his patients. In the 1938 paper translated by Falk, Asperger, in his own words, referred to children ‘whose abnormity is *not of a type that would call for sterilization*, who would socially fail without our understanding and guiding assistance, but who with this help are able to occupy their place in the large organism of our people’ (Asperger 1938). Here, as in all the papers that Falk quotes as evidence for Asperger’s alleged campaign, children with abnormities so severe as to warrant forced sterilization or even death by ‘euthanasia’ are either explicitly excluded (as in this example) or tacitly omitted by using terms like ‘neuropaths,’ ‘psychopaths,’ or ‘abnormal children.’

In my paper, I demonstrate that curative pedagogy was not per se incompatible with Nazi ideology. Asperger’s arguments in favor of the role *Heilpädagogik* (curative pedagogy) could play in salvaging as many children as possible for the community (including, as Asperger explicitly stated, for the Nazi party and the war effort, Asperger 1941) were much less original or offensive to the regime than Falk claims. If the Nazis had seen Heilpädagogik as fundamentally opposed to their designs, they would have closed Asperger’s ward, or would at least not have rewarded him with an academic career. Any meaningful argument, therefore, must focus on the stance that Asperger took vis-à-vis the ‘hopeless’ cases such as (in Asperger’s view) Elisabeth Schreiber and Hertha Schreiber. Falk’s paper not only ignores this entire line of argument, it systematically misrepresents Asperger’s public statements as referring to ‘disabled children,’ including by misleadingly adding the expression ‘[with disabled children]’ to a direct quote from one of Asperger’s papers, where no such reference can be found (Falk 2019: Appendix 2, p. 13; Asperger 1942). In reality, Asperger’s positive remarks (his alleged ‘campaign’) only ever explicitly referred to ‘abnormal’ or ‘difficult’ children, never to the children with severe mental disabilities who were targeted by the ‘child euthanasia’ program implemented at Spiegelgrund. Tellingly, the one publication by Asperger that specifically deals with the condition he attributed to Herta and Elisabeth, ‘postencephalitic’ brain damage, which he deemed often hopeless, is not mentioned in Falk’s paper (Asperger 1944b).

Heilpädagogik’s self-professed mission of turning as many ‘difficult’ children into useful members of the community as possible was—as is demonstrated not least of all by Asperger’s unhindered career—perfectly compatible with a program to murder those whose conditions were too severe to allow such rehabilitation. There is simply no basis for the claim that Asperger campaigned on behalf of children with severe disabilities, who were precisely those in the crosshairs of the ‘euthanasia’ program.

Falk’s paper is similarly misleading on other points, too. For example, her depiction of *Bund Neuland*, which in Asperger’s own words had a lifelong determining influence on his worldview, is extremely one-sided and omits the well-documented fact that the Bund’s vision of a wholesome life was based on an identification of Jews with the ills of modern society, that it formed a bridge between the far-right segments of Austrian Catholicism and the Nazi movement, and that it was infiltrated on all levels by Nazis, including its leader Anton Böhm, who was an agent of the Munich Gestapo (Czech 2018, p. 5–7). When it comes to Asperger’s memberships in Nazi organizations, Falk mentions—correctly—that Asperger never joined the Nazi Party itself, but omits the equally relevant fact that he became, among other things, a candidate of the National Socialist German Physicians League, a subsidiary of the Nazi Party (Czech 2018, p. 9).

Furthermore, my detailed analysis of Asperger’s patient files is misleadingly dismissed as a ‘second-guessing’ of Asperger’s diagnoses, when in reality the chosen method of comparing Asperger’s assessments to those of his contemporaries at Spiegelgrund is designed precisely to avoid the problems associated with a retrospective reevaluation (the comparison incidentally casts severe doubts on Asperger’s self-professed ‘pedagogical optimism’) (Czech 2018, p. 25–28). Regarding Asperger’s approach towards girls, Falk resorts to general assertions of Asperger not being ‘sexist,’ refusing to engage with the documentation I provided of how Asperger’s gendered bias disadvantaged girls in terms of sexual abuse and ‘precociousness’ (Czech 2018, p. 14, 27, 29). Falk’s overall charge against my paper of one-sidedness completely disregards the fact that it discusses in detail relevant historical facts and sources for both sides of the argument. While her paper does not contain a single relevant piece of new evidence, large parts of her argument—for example with regards to Asperger’s apparent reluctance to report his patients for forced sterilization—actually rely on evidence provided in my paper, all the while ignoring the elements that do not support her narrative (Czech 2018, p. 18–9).

Asperger’s case is a complex one, and it deserves a thorough debate without either denial or vilification—a debate that goes beyond the black-and-white dichotomy of hero versus villain. Falk’s paper, with its numerous factual errors, mistranslations, and distortions of the actual content of source documents, does a disservice to this debate. The paper should never have passed peer review and, in view of the academic credibility of all parties concerned, it should be retracted.

## Footnotes

1 Two examples from the first page alone: Franz Hamburger was not the ‘board’ of the Vienna University Clinic, but its director or chairman; and Falk mistook ‘fördern’ in the original as ‘fordern’ (a different word) and thus erroneously translated this as ‘demand,’ where Asperger in fact called to (thus actively) ‘promote’ hereditary health.

2 Municipal and Archives Vienna (WStLA), 1.3.2.209.10, Nervenklinik für Kinder, B 4 Totenbuch.

3 WStLA, 1.3.2.209.10, Nervenklinik für Kinder, Krankengeschichten: verstorbene Mädchen und Knaben 1940–1945, Krankengeschichte Herta Schreiber.

4 In the document, ‘ehesten’ is followed by an ‘s’ and a ‘d’ superimposed on each other, which is a typo.
